# Searching for Information on the Risks of Combined Hormonal Contraceptives on the Internet: A Qualitative Study Across Six European Countries

**DOI:** 10.2196/10810

**Published:** 2019-03-18

**Authors:** Paula Gomes Alves, Irene Petersen, Fiona Stevenson

**Affiliations:** 1 eHealth Unit Research Department of Primary Care and Population Health University College London London United Kingdom; 2 Department of Primary Care and Population Health University College London London United Kingdom

**Keywords:** oral combined hormonal contraceptives, venous thromboembolism, risks, information sources, internet, health information, qualitative research, content analysis

## Abstract

**Background:**

Searching for health information online is increasingly common and is an obvious source of information about oral combined hormonal contraceptives (CHCs) and their risks. However, little is known about how publicly available websites address the risks of CHCs, particularly venous thromboembolism (VTE).

**Objective:**

The aim was to explore the information available to women about VTE and other risks of CHCs on websites available through commonly used search engines.

**Methods:**

A qualitative study was conducted to explore whether and how websites about CHCs in Denmark, Germany, Netherlands, Slovakia, Spain, and the United Kingdom make reference to VTE and other CHC risks. A systematic search procedure was adopted across the six countries, based on relevant keywords. The search was carried out using the Google search engine by fluent/native speakers of each language. A content analysis approach was conducted to extract information from the selected websites.

**Results:**

A total of 357 websites were reviewed. Nearly all (343/357, 96.1%) the websites mentioned VTE as a risk of CHCs, with approximately half referring to other side effects as well. One-fifth (92/357, 25.8%) of the websites provided suggestions about the best contraceptive method to use, and only a minority (23/357, 6.4%) recommended women discuss CHCs with their health professionals. Sites were generally run by the media (110/357 30.8%) or medical services from nongovernmental organizations (140/357, 39.2%). Only a minority of websites referred to organizations such as the European Medicines Agency (11/357, 3.1%).

**Conclusions:**

Despite the large number of websites containing information about oral CHCs and their risks, particularly VTE, only a limited number referred to information from accredited health agency sources. We argue this is a missed opportunity for accredited health agencies to share high-quality information to assist women using CHCs to make informed decisions about contraception.

## Introduction

Women using combined hormonal contraceptives (CHCs), which contain both estrogen and progesterone, may be three times more likely at risk of venous thromboembolism (VTE) compared to female nonusers [1]. VTE is a condition that involves formation of a blood clot in a vein and can lead to various health complications (eg, breathing difficulties, chest pain), some of which are life-threatening [2].

According to the European Medicines Agency (EMA), the overall risk of VTE is small and “the benefits of CHC in preventing unwanted pregnancies continue to outweigh their risks” [3]. In their 2014 review, the EMA emphasized that women should be provided with updated information about CHC risks, particularly VTE, so that an informed decision about contraception can be made [4]. It is unclear how women are being provided with this information when prescribed CHCs. A European Union-wide survey conducted by the European Commission in 2014 reported that most Europeans (>75%) believe that the internet is a good resource for health-related information, with 6 in 10 people reporting they “go online when looking for health information” [5]. Therefore, it is likely that women are using the internet to search for information about CHCs. However, little is known about the availability and the type of information that women can find online about CHC-related VTE and other risks of this contraceptive method.

According to the most recent statistics (2017), approximately 90% of females in all EU countries aged 16 to 29 years have used the internet in the last 3 months [6]. The reasons for using the internet are many and may vary between individuals. Similarly, in the 2014 European Commission study, 8 in 10 people aged 15 to 39 years reported using the internet as a health information resource [5]. A recent study by Haluza et al [7] reported that 79% of people referred to the internet as a primary source of health information, outnumbering people using sources such as the doctor (74%), books (63%), or family (62%).

Understanding the information available to women about CHCs and their associated risks is a pressing issue, particularly because previous literature has suggested that women may have knowledge gaps and misconceptions about contraceptives. Previous research has shown that providing women with information about CHC not only improves their knowledge and awareness of risks but also allows them to make a more informed choice [8-10]. Studies have also reported that receiving information about contraception has encouraged women to opt for alternative methods than those they had initially considered [9,10].

It is important to explore the type of information about CHCs and risks available when searching online via common search engines. The goals of this study were two-fold: (1) to identify European websites focusing on oral CHCs and their associated risks, particularly VTE, available through commonly used search engines; and (2) to explore the information provided about CHCs and their risks provided on these websites through a content analysis approach. This study was part of a larger project [11] exploring how women and health professionals communicate about the risks of using CHCs, the process by which women decide to use a CHC, and the information sources that prescribers and women rely on to support their decisions.

## Methods

### Overview

In this qualitative study we identified websites that provided information about CHCs available through common search engines and analyzed the information extracted from the websites about the risks associated with the use of CHCs. Websites were systematically identified across six countries in Europe: Denmark, Germany, Netherlands, Slovakia, Spain, and the United Kingdom. The selection was made to ensure geographical coverage of southern, central, eastern, northern, and noncontinental European regions.

### Development of the Search Protocol

A protocol was developed in English to ensure consistency across the online searches for websites about CHCs across the six countries. Three English lay advisors were consulted for which keywords they would use to search for CHCs and their risks. The three lay advisors were women aged 20 to 30 years. Each woman was asked, “Which words would you use to search for information online about the risks of blood clots when taking the pill?” This led to the identification of keywords that were meaningful and relevant to women (lay terms) and to, as far as possible, ensure that our search would retrieve results that were equivalent to those obtained by women when searching for information about oral CHCs. English-speaking women were used because the protocol was developed in English.

**Figure 1:**
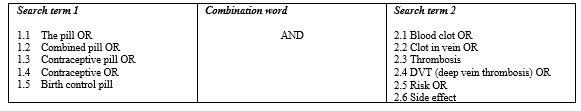
Keywords and the search strategy used to search for combined hormonal contraceptives.

The keywords generated by the three laywomen advisors were then discussed by the research team, who added relevant synonyms to ensure a larger coverage of results (see Figure 1).

After defining the keywords, we moved on to produce a step-by-guide guide on how to perform the online searches. A preliminary version of this search protocol was then revised and piloted by another laywoman who was a native speaker of English; she commented on the clarity and the appropriateness of the language used. The final version of the protocol was delivered in March 2017 and disseminated to the four women recruited to conduct the searches.

### Search and Selection of Websites

The search for websites about CHCs was carried out using the Google search engine in each country. We opted for Google because it is considered to be one of the most popular search engines. Google was also used in previous studies about women-related health issues [12].

**Inclusion and exclusion criteria for website selection about combined hormonal contraceptives (CHCs).**

**Inclusion criteria**

- Websites targeting women and/or health professionals
- Provides information about oral CHCs
- Contains discussion of the risks of CHCs, including venous thromboembolism
- Updated since the latest guidance from the European Medicines Agency was published (2013)

**Exclusion criteria**

- Scientific electronic databases with links to publications about this topic
- No reference to oral CHCs and/or their risks
- Non-European websites
- Not updated since the latest guidance from the European Medicines Agency was published (2013)

Due to the large number of results available on Google, we limited our search to the first 10 pages of results. We limited the page results because previous studies have shown that when it comes to online reviews of products, consumers rely on a very limited number of reviews to make their decisions [13]. The limit of 10 pages was chosen by the research team in consultation with another laywoman advisor independent to the study, who reported that in real-life searches it is unlikely that people will search further than this. The websites retrieved from each country were selected according to the criteria detailed in Textbox 1.

### Search Procedure and Data Extraction

We recruited four laywomen via the research team network to conduct the Google searches. We opted for laywomen, as opposed to researchers, to conduct the searches because we wanted to replicate how women search for CHCs in naturalistic settings. Also, previous research has suggested that contraceptive responsibilities tend to lay primarily with women [14], making it more likely that searches about CHCs are conducted by women rather than men.

We recruited women of child-bearing age who were native or fluent English speakers, and also native or fluent speakers of at least one of the other five languages of the countries included in the study (see Table 1). We opted for women who were fluent in English to ensure that the same protocol could be used consistently across the team.

Before starting the search, all women were instructed to translate the keywords into their own language. Because the women were considered laypeople, they were instructed to adapt the English keywords to what they would mean in their language. This ensured that all non-English keywords used represented the lay terms that women would potentially rely on to search in the other non-UK countries. The research team discussed these translations with women to ensure they were equivalent to the English terms and that they represented lay, relevant terms in each country.

The internet search was independently conducted by the women in March 2017. They were all based in London; therefore, the computers used for the searches were connected to a virtual private network (VPN) service for remote access to local/national internet servers. This ensured the retrieval of results were equivalent to those obtained by individuals based in each country.

Once connected to the VPN service, women were instructed to open their local version of Google (eg, for Spain, www.google.es) and enter the keywords in the “Search” field. The search involved combining all the variations of search term 1 with each of the six variations of search term 2 (eg, “the pill OR combined pill OR contraceptive OR contraceptive pill OR birth control pill AND blood clot”), resulting in a total of six searches (see Figure 1). To avoid the identification of websites using the languages of interest, but whose origin was not in the countries under study (eg, a website written in German targeting Austria or Switzerland), women were also instructed to use the “Advanced search” menu on Google and to narrow the results to “Country/Region” (Denmark, Germany, Netherlands, Slovakia, Spain, and the United Kingdom) and “Site/Domain” (.dk, .de, .nl, .sk, .es, and .uk).

**Table 1:** Sociodemographic and language background of the women (N=4) who conducted the internet searches about combined hormonal contraceptives (CHCs).

Searcher	Age range	Education^a^	Employment	Previous user of oral CHC	Languages
Woman 1	25-34	Postgraduate education	Part-time	Yes	English (fluent); Spanish (fluent)
Woman 2	18-24	A-levels/GCSE	Student	No	English (native); Danish (native); German (fluent)
Woman 3	35-44	A-levels/GCSE	Part-time	Yes	English (fluent); Slovakian (native)
Woman 4	35-44	Postgraduate education	Full-time	Yes	English (fluent); Dutch (native)

^a^GCSE: General Certificate of Secondary School, equivalent to high school in the United Kindgom

### Data Analysis

After the selection of websites in the first 10 pages of results, a standardized Microsoft Excel spreadsheet was created for data extraction (Multimedia Appendix 1). This spreadsheet was used by women to extract information about each website found in their respective countries. Women were instructed to complete the data extraction form in English. The data extraction form included both close-ended questions, which focused on general website characteristics, and an open-ended question for women to describe, in free text, which information was contained on the website. We summarized the women’s responses to the close-ended items to gain an overview of the structure of the websites through a basic descriptive analysis (eg, counts and percentages).

However, the focus of our study was the qualitative information provided by the women when describing the websites’ contents. These qualitative data were analyzed using a content analysis approach [15]. We started with a preliminary reading of the women’s descriptions of the websites, from which categories of information emerged. These categories represented the content, or the topics covered, by the website. After developing this categorical system, the women’s descriptions were revisited, and each website was assigned to categories according to the information they covered.

The first author conducted both the definition of the categories and the categorization of the websites. For quality control purposes, the development of the categories was discussed with a senior researcher with expertise in qualitative research. Then, we randomly selected 10% of the websites and invited one of the women who assisted with the internet search to discuss the categorizations made by the first author. For this process, we asked the woman to consider the categories assigned to each website and consider whether she agreed with the categorization. After discussion, the categorizations were readjusted, and the first author proceeded with the categorization of the remaining websites.

## Results

### Overview of the Website Characteristics

In total, 357 websites with information about oral CHCs were selected across the six countries. The majority of these were provided by either medical services from nongovernmental organizations (NGOs) (140/357, 39.2%) or media (110/357, 30.8%), including newspapers and online magazines. See Table 2 for detailed information on the types of websites with information about CHC-related VTE and other risks organized according to the country of origin. Websites tended to mostly target women (237/357, 66.3%), with a few focusing on particular subpopulations such as young women or students (4/357, 1.1%). One-fifth of the websites (81/357, 22.6%) did not refer to a specific group and, therefore, were considered to be targeting the general population. Half of the websites selected (185/357, 51.8%) were updated between 2015 and 2017. However, one-fifth of the websites (89/357, 24.9%) did not make any reference to when their website had last been updated.

Websites in Denmark, the Netherlands, and Slovakia did not make reference to the EMA guidelines, with mentions in the other countries minimal, namely Germany (3/113, 2.7%), Spain (1/22, 4.5%), and the UK (7/80, 8.8%). Few websites were interactive, for example with the provision for users to post questions and discuss topics with other individuals. The exceptions were websites in Spain (12/22, 54.5%) and the UK (34/80, 42.5%).

### Contents Covered by Websites Focusing on Combined Hormonal Contraceptives and Their Risks

Twenty themes emerged from the content analysis of the free-text website descriptions provided by the women across the six countries. A description of these themes and their respective theme codes used to categorize the information covered by each website are provided in Textbox 2.

Having developed this schema of 20 themes, we categorized each of the 357 websites and explored the type of information that was covered by those websites. The results of this process are presented in Table 3.

**Table 2:** Distribution of websites per country according to their type (N=357).

Type of website	Country, n (%)						
	All countries (N=357)	Denmark (n=82)	Germany (n=113)	Netherlands (n=38)	Slovakia (n=22)	Spain (n=22)	UK (n=80)
Beauty/fitness/lifestyle website	25 (7)	6 (7)	15 (13)	0 (0)	1 (5)	2 (9)	1 (1)
Charity	6 (2)	0 (0)	1 (1)	3 (8)	0 (0)	0 (0)	2 (3)
Health blog/network	26 (7)	4 (5)	5 (4)	2 (5)	3 (14)	4 (18)	8 (10)
Insurance services	1 (0)	0 (0)	0 (0)	1 (3)	0 (0)	0 (0)	0 (0)
Legal services	2 (1)	0 (0)	1 (1)	0 (0)	0 (0)	0 (0)	1 (1)
Media	110 (31)	16 (20)	42 (37)	1 (3)	0 (0)	9 (41)	42 (53)
NGO medical services	140 (39)	54 (66)	46 (41)	7 (18)	18 (82)	2 (9)	13 (16)
National health service	5 (1)	0 (0)	0 (0)	1 (3)	0 (0)	1 (5)	3 (4)
Personal blog	4 (1)	2 (2)	0 (0)	0 (0)	0 (0)	1 (5)	1 (1)
Petition website	1 (0)	0 (0)	1 (1)	0 (0)	0 (0)	0 (0)	0 (0)
Religious blog	1 (0)	0 (0)	0 (0)	0 (0)	0 (0)	0 (0)	1 (1)
Science blog	2 (1)	0 (0)	1 (1)	0 (0)	0 (0)	0 (0)	1 (1)
Support group	33 (9)	0 (0)	1 (1)	23 (61)	0 (0)	3 (14)	6 (8)
Travel blog	1 (1)	0 (0)	0 (0)	0 (0)	0 (0)	0 (0)	1 (1)

**Themes (categories) covered by websites on combined hormonal contraceptives, including their codes and definitions/explanations.**

**ADP:** Advantages/benefits about the pill

**AP:** Alternative contraceptive methods to using the pill

**BC:** Blood clots as risk

**BWER:** Bad/negative women experiences or reports about using the pill

**DC:** Doctor communication (ie, the importance of talking with doctor about the pill)

**DP:** Disadvantages about the pill

**EA:** Emergency advice (eg, warning symptoms, life-saving actions)

**EB:** Emphasis on beauty/lifestyle motives for using the pill (eg, improving skin, hair)

**FC:** Family communication (ie, the importance of talking to family members, in particular mothers, about the pill)

**GIP:** General information about the pill (eg, history, how it works, how to take it)

**IC:** Information about contraceptive methods in general

**MP:** Myths about the pill

**NBC:** No blood clots mentioned as a risk

**RD:** Resources for doctors (eg, symptom checklists, procedures to adopt)

**RV:** Risk variations when using the pill (eg, groups with higher risk, risk factors)

**SPD:** Suggests/advises pill is dangerous to use

**SPS:** Suggests/advises pill is safe to use

**VSE:** Various side effects of the pill (besides blood clots)

**WP:** Which is the most dangerous/safe type of pill

**WW:** Women worries about the pill

**Table 3:** Themes covered by the selected websites across the six countries (ranked by frequency for all countries).

Themes^a^	Country, n (%)						
	All countries (N=357)	Denmark (n=82)	Germany (n=113)	Netherlands (n=38)	Slovakia (n=22)	Spain (n=22)	UK (n=80)
BC	343 (96)	78 (95)	113 (100)	37 (97)	19 (86)	22 (100)	80 (100)
VSE	164 (46)	37 (45)	112 (99)	20 (53)	6 (27)	13 (59)	77 (96)
GIP	151 (42)	53 (65)	33 (29)	20 (53)	12 (55)	8 (36)	27 (34)
RV	121 (34)	41 (50)	109 (96)	4 (11)	5 (23)	2 (9)	16 (20)
WP	92 (26)	41 (50)	40 (35)	2 (5)	1 (5)	0 (0)	8 (10)
ADP	80 (22)	26 (32)	24 (21)	16 (42)	0 (0)	2 (9)	12 (15)
EA	65 (18)	26 (32)	23 (20)	5 (13)	2 (9)	0 (0)	9 (11)
BWER	62 (17)	9 (11)	17 (15)	10 (26)	0 (0)	0 (0)	70 (88)
DP	54 (15)	11 (13)	23 (20)	12 (32)	1 (5)	1 (5)	6 (8)
AP	41 (11)	13 (16)	22 (19)	0 (0)	1 (5)	0 (0)	5 (6)
SPS	35 (10)	18 (22)	14 (12)	1 (3)	0 (0)	0 (0)	2 (3)
IC	26 (7)	11 (13)	2 (2)	1 (3)	0 (0)	9 (41)	3 (4)
DC	23 (6)	5 (6)	13 (12)	0 (0)	0 (0)	0 (0)	5 (6)
SPD	21 (6)	8 (10)	9 (8)	0 (0)	1 (5)	1 (5)	2 (3)
EB	18 (5)	0 (0)	17 (15)	0 (0)	0 (0)	0 (0)	1 (1)
NBC	10 (3)	3 (4)	4 (4)	1 (3)	3 (14)	0 (0)	0 (0)
MP	9 (3)	1 (1)	1 (1)	2 (0)	0 (0)	1 (5)	4 (5)
RD	3 (1)	0 (0)	3 (3)	0 (0)	0 (0)	0 (0)	0 (0)
WW	2 (1)	0 (0)	0 (0)	0 (0)	0 (0)	0 (0)	2 (3)
FC	1 (0)	1 (1)	0 (0)	0 (0)	0 (0)	0 (0)	0 (0)

^a^ADP: Advantages/benefits about the pill; AP: alternative contraceptive methods to using the pill; BC: blood clots as risk; BWER: bad/negative women experiences or reports about using the pill; DC: doctor communication (ie, the importance of talking with doctor about the pill); DP: disadvantages about the pill; EA: emergency advice (eg, warning symptoms, life-saving actions); EB: emphasis on beauty/lifestyle motives for using the pill (eg, improving skin, hair); FC: family communication (ie, the importance of talking to family members, in particular mothers, about the pill); GIP: general information about the pill (eg, history, how it works, how to take it); IC: information about contraceptive methods in general; MP: myths about the pill; NBC: no blood clots mentioned as a risk; RD: resources for doctors (eg, symptom checklists, procedures to adopt); RV: risk variations when using the pill (eg, groups with higher risk, risk factors); SPD: suggests/advises pill is dangerous to use; SPS: suggests/advises pill is safe to use; VSE: various side effects of the pill (besides blood clots); WP: which is the most dangerous/safe type of pill; WW: women worries about the pill.

Nearly all selected websites mentioned VTE as a risk of CHCs (343/357, 96.1%). Just under half of the websites (164/357, 45.9%) mentioned various side effects of CHCs and general information about CHCs (151/357, 42.2%). One-third of websites mentioned risk variations in taking CHCs according to personal characteristics/clinical history (121/357, 33.9%). A quarter of the websites provided suggestions to which oral contraceptive was the best to use (92/357, 25.8%) and the advantages of CHCs (80/357, 22.4%). Only a minority of websites emphasized the importance of talking to health professionals about CHCs (23/357, 6.4%).

## Discussion

To our knowledge, this is the first and largest study to date to qualitatively explore the information available to women searching online for information about CHCs and their risks.

This study aimed to explore online information sources which mention CHCs and their risks, which are currently and publicly available to women across six EU countries. A content analysis approach was used to explore what was covered by these websites. The websites were identified through a systematic, standardized Google search in six languages. We found many websites that provided information about oral CHCs and their risks. There were a limited number of personal blogs providing women’s accounts and experiences with CHCs. Overall, the websites did not offer the possibility for users to ask further questions and share their experiences and rarely included references to health agencies such as the EMA. Most websites included information on VTE and other risks of oral CHCs, but only a minority advised users to seek further information from a health professional.

The fact that information about CHCs is largely provided by media websites and NGO medical services raises questions about the quality of the information provided. As in any other medium, the quality of the information provided across websites is likely to vary, and regulation of quality standards is hard to implement [16]. Rowe et al [17] stated that, in the UK and Sweden, media reports tend to present health risks in an “alarmist rather than reassuring” way, and seldom provide details about statistics to “express degrees of risk.” Almost a decade later, Walsh-Childers et al [18] reported an improvement over the years in the completeness and usefulness of news stories about health, but also a decline in the number of articles reporting health news.

When it comes to information provided by medical services, it is possible that the contents are not always easy to understand from a general population perspective. The concept of patient health literacy has been explored in many other contexts. For instance, in a study by Dickens et al [19], it was suggested that health professionals may overestimate patients’ health literacy. This conclusion was because of 68% of patients whose health literacy was judged as adequate by health professionals, only 22% were considered to be health literate according to the Newest Vital Sign test, which aims to measure low health literacy [19]. In the field of contraception, a study conducted in 2007 reported that the reading level for contraceptive instructions was the sixth to twelfth grade for condoms, ninth to tenth grade for spermicides, and tenth to twelfth grade for emergency contraception, with the twelfth grade corresponding to education attained at 18 years of age. However, according to the latest Eurostat 2017 statistics [20], there are numerous individuals in our countries of interest with “less than primary, primary, and lower secondary education.” Lower education corresponds to education attained at 13 years of age (Denmark 17%, Germany 13%, Netherlands 19%, Slovakia 7%, Spain 37%, and UK 19%). The scenario becomes even more complex when it comes to using health information which was searched online. According to authors such as Norman and Skinner [21], consumers of eHealth information need to have additional literacy skills to explore the data available on websites, which include not only being able to understand the information itself, but also the ability to seek, find, and appraise the quality of the information found. Hence, further research is needed to explore whether both the literacy and eHealth literacy of those searching for online information about CHC and its risks to support contraception choices.

This study also suggests that websites about contraceptive are generally aimed at women. Literature has also referred to contraception as a topic that is socially perceived to be within the “women’s sphere” [14]. If information provided in websites is not gender neutral, this may be perpetuating the idea that contraception is a decision placed on women as opposed to partnerships. Future research should explore the perceptions of males about CHCs, their level of involvement in contraception choices, and which information sources do men seek when it comes to contraception.

Our study demonstrated that most websites were not interactive (ie, did not allow its users to make comments, share experiences, or ask questions about the content provided). Previous literature has demonstrated that the internet, including social media platforms, is becoming an important platform for communication between patients and health professionals. According to Rolls et al [22], health professionals are increasingly using social media to share their knowledge. As a result, several guidelines have emerged about how professionals should use online communication tools to share health information [23,24]. The internet is a powerful source of health information, including topics such as contraception; therefore, professionals may be missing an opportunity to communicate with women about oral CHCs using the internet. We acknowledge that having websites monitored by health regulators or health professionals with active communication features available to their users is unlikely to be feasible. However, we propose that popular health-related websites may develop algorithms which allow them to understand (eg, the information most frequently accessed by website users) and use that as starting point to improve the way in which such information is shared with the target audience.

Despite giving information about the risks of oral CHCs, there are several websites which also advise about the best oral CHC, including references to different brands. This topic has been widely addressed, with many authors referring to “Dr Google” as an expression that reflects the role that the internet has assumed in searching for health information. In the study by Lee et al [25], it was reported that nearly half of individuals with chronic health conditions use Web-based health information to help manage their condition. Individuals seek health information online due to the convenience, coverage, and anonymity [26]. For this reason, the health community must acknowledge the role the internet is playing in helping people making decisions about their health care, and increase involvement with the internet as a prime medium for communication of the latest evidence.

Citation of either European (eg, EMA) or local (eg, National Health Service) health agencies was limited. Variation in reference to health agencies presents the possibility of further involvement in promoting initiatives to raise awareness about the extent of their work among the general population. A possible example for modeling is the project “Decides Espana” [27], which launched in Spain in 2014. This project focused on improving sexual and reproductive rights, and one of its goals was to “raise the awareness of health institutions on the notion of equity in health.” Although this project targeted cultural minorities, it provides an example of initiatives that could be promoted at a local and European level to increase the population knowledge about health organizations that individuals can contact or access to search for reliable and up-to-date information.

This study is not without limitations. First, the search was conducted in different languages, derived from a protocol prepared in English; therefore, it is uncertain whether all key terms used were completely equivalent across countries. We consider the impact of this to be minimal because the women conducting the searches were bilingual or fluent in English. Second, this study only discusses information about CHCs included on the websites and was unable to assess the quality of this information. Third, the six countries included did represent some diversity across Europe, with four of the six being part of Northern Europe, one from Eastern Europe, and one from Southern Europe. However, further research could target other Southern and Eastern European countries. Nevertheless, we consider this study to be a pioneer in its approach of systematically searching for and reviewing health information which is readily and freely available to all internet users. We hope this might encourage other researchers to focus on evaluating the quality of health-related information provided online. This is particularly important when it comes to using oral CHCs. CHCs have been marketed as a “lifestyle drug” (ie, “medications to improve a person’s quality of life”), which may also have a “cosmetic, life-enhancing, recreational, or discretionary” value [28], yet carries a small but important potential risk to health through VTE, which may not be evident among the normalization of use of CHCs.

In conclusion, this study has shown that there are numerous online information sources about oral CHCs. It is crucial that regulatory health agencies and health professionals engage in this space to inform potential and current users of CHCs both inside and outside the consultation room. In a digital era, and when there is little control over the contents that internet users post online (eg, personal blogs), such active involvement by health authorities would allow for CHC users to be able to make fully informed decisions about their contraceptive needs.

## Appendix

Multimedia Appendix 1 Data Extraction Form (Table format).

## Abbreviations

CHC: combined hormonal contraceptives
EMA: European Medicines Agency
NGO: nongovernmental organizations
VTE: venous thromboembolism
