# Supplementary material for: Searching for Information on the Risks of Combined Hormonal Contraceptives on the Internet: A Qualitative Study Across Six European Countries
Source: J Med Internet Res. 2019 Mar 18;21(3):e10810. doi: 10.2196/10810 (PMC6441861; doi:10.2196/10810)
Supplement: Multimedia Appendix 1 [file jmir_v21i3e10810_app1.pdf]

Appendix 1

Data extraction form and brief explanation of its content

Variable	Sub-Variable	Definition
Name of host website	-	Title/designation of the website
Website address	-	Hyperlink to the website
Target population	General population	All population, without specifying a particular group
	Health professionals	Only health professionals including doctors, nurses or any health-related profession
	Under 25s/youth/students	Youth population, including adolescents and students
	Women	Only women
	Women/health professionals	Both women and health professionals
Type of website	Beauty/fitness/lifestyle website	Online magazines or blogs focusing on women beauty, fitness and lifestyle
	Charity	Websites run by/representing charity organisations
	Health blog/network	Blog containing health advice and/or information
	Insurance services	Websites advertising/representing insurance companies
	Legal services	Law/legal advice and consultations
	Media	Newspapers, magazines and TV channels
	NGO medical services	Non-governmental medical groups and/or services providing clinical support
	National health service	Governmental departments about healthcare
	Personal blog	Individual blogs expressing personal/independent views
	Petition website	Websites advertising for online petitions
	Religious blog	Websites run by/representing religious groups
	Science blog	Websites reporting and/or discussing scientific news
	Support group	Websites run by/representing support groups

	Travel blog	Blogs providing health advice and information for travellers
Brief description of information provided	-	Summary of the contents covered by the website (e.g. headings, main message, etc.)
Last updated to website	-	Year
References/data sources	-	Cited sources, such as guidelines, papers or national and international organisations
Existence of forums/discussion groups	-	Websites containing message boards, discussion groups or links to social networks
